# Sir Spencer Wells's *Abdominal Tumours and Revival of Ovariotomy*

**Published:** 1885-06

**Authors:** 


*Diagnosis and Surgical Treatment of Abdominal Tumours.* By Sir SPENCER WELLS, Bart. Pp. 216. London: J. & A. CHURCHILL. 1885.

*The Revival of Ovariotomy.* By Sir SPENCER WELLS, Bart. Inaugural Address read before the Midland Medical Society. 1885.

IN the face of some recent controversy as to whom merit is chiefly due in establishing Ovariotomy among justifiable surgical operations, it is a significant fact that the Midland Medical Society should have invited Sir Spencer Wells to address them in Birmingham. From Birmingham chiefly have arisen the protests against the claims of Sir Spencer Wells to this position, and we can scarcely avoid the conclusion that this Address was intended to be reckoned either as a defence of an assailed position, or as a pæan after an accomplished triumph.

If it be not true that Sir Spencer Wells has insufficiently recognised the claims of Charles Clay, of Manchester, it is clear enough that he has simply ignored the work of Lawson Tait, of Birmingham. Most of us would have supposed that no work on the surgery of abdominal tumours would be complete without some reference to the labours of Tait: Sir Spencer Wells does not even mention his name.

We have always considered that Clay has not received justice at the hands of Wells. In 1873, Peaslee, of New York, a writer to whom neither bias nor want of knowledge can be ascribed, thus wrote of Clay's work: "To Dr. Charles Clay, of Manchester, however, more than to all other operators, the credit belongs of having placed the operation of ovariotomy on a sure foundation. Fehr calls him 'the original herb' of this operation. . . . . He at length overcame, in a great degree, the opposition in England to ovariotomy, by his fairness in reporting his cases, his scholarship, and especially by his success." Up to 1850, eight years before the date of Wells' first operation, he had operated thirty-three times with twenty-one successes, a mortality less than Wells had in his first thirty-three, not finished till twelve years later. Wells complains that Clay did not sufficiently make public his cases. He certainly did not publicly say in the weekly journals that he wished to be an ovariotomist, and that he would faithfully publish the results of all the cases his brethren might send him; but he made no secret of his work. He contributed more than one hundred papers to various periodicals, and wrote several large professional works. As far as the labours of a mere man can go, surely this was enough. Many able scientific men are too modest and retiring to put themselves forward as pioneers in any walk. They set about their work quietly and silently, because they do not court publicity; they are late in reaping their rewards, or even die unappreciated. Sir Spencer Wells has, of a surety, reaped his reward while yet alive. We are not sure that Wellington would have added to his fame by the delivery of a lecture to prove that he was a great warrior; and we scarcely think that Wells has added to his repute by this Address, proving himself a great ovariotomist. Amidst all this talk our mind wanders to the Northern Metropolis, where another ovariotomist, greater than them all in daring and in success, has failed to reap the titles and rewards which were undoubtedly his due, simply because he has been too silent, from too much modesty.

We have as little sympathy with those who seek to minimise the reputation of Wells, as with those who seek to exaggerate it. If much of his practice has been bad, it has abundantly taught us to avoid it; if any of it has been good, we have been fully informed of it. In either case, we must regard him as a benefactor to our race.

Of the book itself, little need be said. It is essentially a fourth edition, much cheapened, of his well-known work. Some chapters have been suppressed, and new chapters on new operations have been added. The value of these new chapters is much diminished by the writer having completely ignored the work of Tait. Lastly, we confess we do not like the tone of the preface.

